# The Notch pathway promotes the cancer stem cell characteristics of CD90^+^ cells in hepatocellular carcinoma

**DOI:** 10.18632/oncotarget.6672

**Published:** 2015-12-19

**Authors:** Jing Luo, Peng Wang, Ronghua Wang, Jinlin Wang, Man Liu, Si Xiong, Yawen Li, Bin Cheng

**Affiliations:** ^1^ Department of Gastroenterology and Hepatology, Tongji Hospital, Tongji Medical College, Huazhong University of Science and Technology, Wuhan 430030, P.R. China; ^2^ Department of Emergency, Tongji Hospital, Tongji Medical College, Huazhong University of Science and Technology, Wuhan 430030, P.R. China

**Keywords:** Notch pathway, HCC, CD90, cancer stem cells

## Abstract

CD90 has been identified as a marker for liver cancer stem cells (CSCs) that are responsible for tumorigenic activity, but it is not known how CD90^+^ cells contribute to tumor initiation and progression. Our data demonstrated that high expression of CD90 in Hepatocellular Carcinoma (HCC) tissues correlated with venous filtration in HCC patients. CD90^+^ cells isolated from HCC cell lines exhibited increased tumorigenicity, chemoresistance, tumor invasion and metastasis. Notch pathway was activated in CD90^+^ cells and we found that inhibition of Notch pathway in CD90^+^ CSCs decreased tumorigenicity, cell invasion, migration and expression of stem cell related genes. Activation of Notch pathway in CD90^−^ cells induced self-renewal, invasion and migration. Furthermore, we observed that cancer stem cell features were facilitated by stimulating G1-S transition in the cell cycle phase and inhibiting apoptosis mediated by Notch pathway. Our findings suggested CD90 could be used as a potential biomarker for HCC CSCs, and that cancer stem cell activity was elevated through up activated Notch pathway in CD90^+^ CSCs.

## INTRODUCTION

Liver cancer is the fifth most commonly diagnosed cancer and the second most frequent cause of cancer death in men worldwide [1]. There is increasing evidence that resistance to HCC therapy is, at least in part, caused by inherent resistance of a subpopulation of cancer cells. This subpopulation shares many properties with stem cells and thus has been labeled as CSCs [2, 3]. CSCs are highly tumorigenic, metastatic, chemotherapy and radiation resistant, responsible for tumor relapse after therapy, and they are also able to divide symmetrically and asymmetrically to orchestrate tumor development and progression [4]. Using a variety of stem cell markers, CSCs have been identified in solid tumors, including pancreatic cancer, colon cancer, breast cancer and HCC [4-7]. Although many CSC biomarkers (e.g., CD133, CD13, CD24, EpCAM, Nanog) have been identified in HCC, it is still unclear which biomarker truly represents CSCs and the molecular signaling events that regulate cellular hierarchy, stemness, and success in defining key CSC-specific genes [8-11].

CD90 (Thy-1) is a 25-37 kDa glycosylphosphatidylinositol (GPI)-anchored glycoprotein expressed mainly in leukocytes, and is involved in cell-cell and cell-matrix interactions [12]. Zhen et al.'s study reported that CD90^+^ cells, but not CD90^−^ cells, from HCC cell lines displayed tumorigenic capacity [13]. In our study CD90^+^ cells not only possessed high tumor formation ability, but also other features of cancer stem cells such as extensive proliferation, differentiation, chemoresistance, tumor invasion and metastasis.

The Notch signaling pathway is an evolutionarily conserved pathway and has been reported to promote the self-renewal, differentiation, proliferation, survival, angiogenesis, and migration of CSCs in several malignancies [14, 15]. It is one of the most intensively studied candidate therapeutic targets in cancer stem cells, and several Notch inhibitors are being developed [16-18]. Zhen et al.'s study reported CD90^+^ cells isolated from normal and cirrhotic livers, tumor tissues, and blood samples of HCC patients expressed a comparable level of Notch1 with CD90^−^ cells [13]. Whether Notch signaling pathway was involved in the activation of cancer stem cell features of CD90^+^ cells remained ambiguous.

## RESULTS

### High CD90 expression in HCC clinical specimens was associated with venous infiltration and poor prognosis

**Figure 1:**
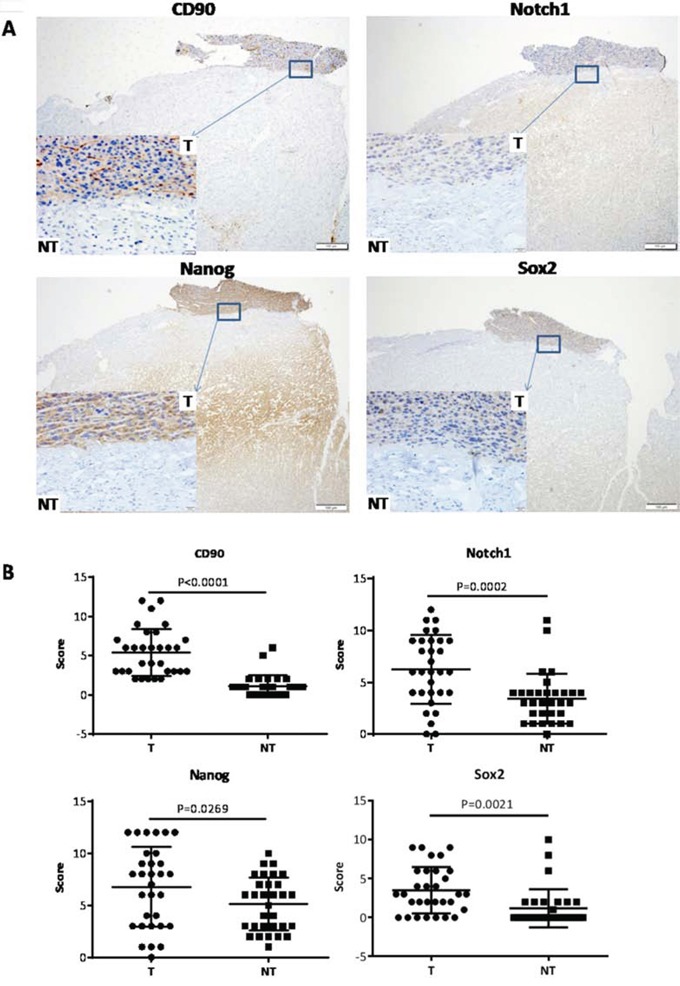
Expression of CD90, Notch1, Nanog, Sox2 in HCC and non-HCC tissues **A.** Representative microphotograph of CD90, Notch1, Nanog and Sox2 staining in HCC tissues (T) and their paired surrounding non-HCC tissues (NT) by IHC analysis. **B.** Analysis of expression of CD90, Notch1, Nanog and Sox2 in HCC and non-HCC tissues by paired t test.

31 pairs of human HCC and their paired corresponding non-HCC tissues were collected from hepatic surgery at Tongji Hospital. The expression levels of CD90, Notch1, Nanog and Sox2 (stem cell related genes) were evaluated in parenchymal hepatic cells (excluding mesenchymal and vascular endothelial cells) by IHC and found to be significantly overexpressed in HCC (Fig. 1A, 1B). Data analysis was performed using clinical history information of 31 HCC patients and CD90 expression level (Table 1). There was no significant correlation between CD90 expression and these clinical factors, such as age, tumor size, TNM stage, microsatellites, serum AFP level and differentiation status. Interestingly, four out of five patients with high CD90 expression correlated significantly with venous infiltration (P < 0.0001, Fisher's exact test). Two of those four patients have developed HCC recurrence, of the other 26 patients with low CD90 expression only occured 5 cases of recurrence and metastasis. These results indicated that high CD90 expression which may correlate with poor prognosis of HCC patients.

**Table 1:** The correlation between clinical pathological information and CD90 expression in HCC patients

Clinical-Pathological Variables	Low CD90 (N=26)	High CD90 (N=5)	p Value
Venous Infiltration Absence Presence	- 26 0	- 1 4	<0.0001*
Serum AFP Level Low(≤20 ng/ml) High(>20 ng/ml)	- 6 20	- 1 4	1
TNM Stage** Early Stage(I-II) Advanced Stage(III-IV)	- 22 4	- 1 4	0.2406
Microsatellites Absence Presence	- 26 0	- 4 1	0.1613
Age Young(≤median,47) Old(>median,47)	- 13 13	- 3 2	1
Differentiation Status Well differentiated Moderately to poorly differentiated	- 5 21	- 0 5	0.5601
Tumor Size Small(≤5cm) Large(>5cm)	- 6 20	- 1 4	1

* Significant difference

** AJCC/UICC T staging system

### CD90^+^ HCC cells exhibited characteristics of cancer stem cells

**Figure 2:**
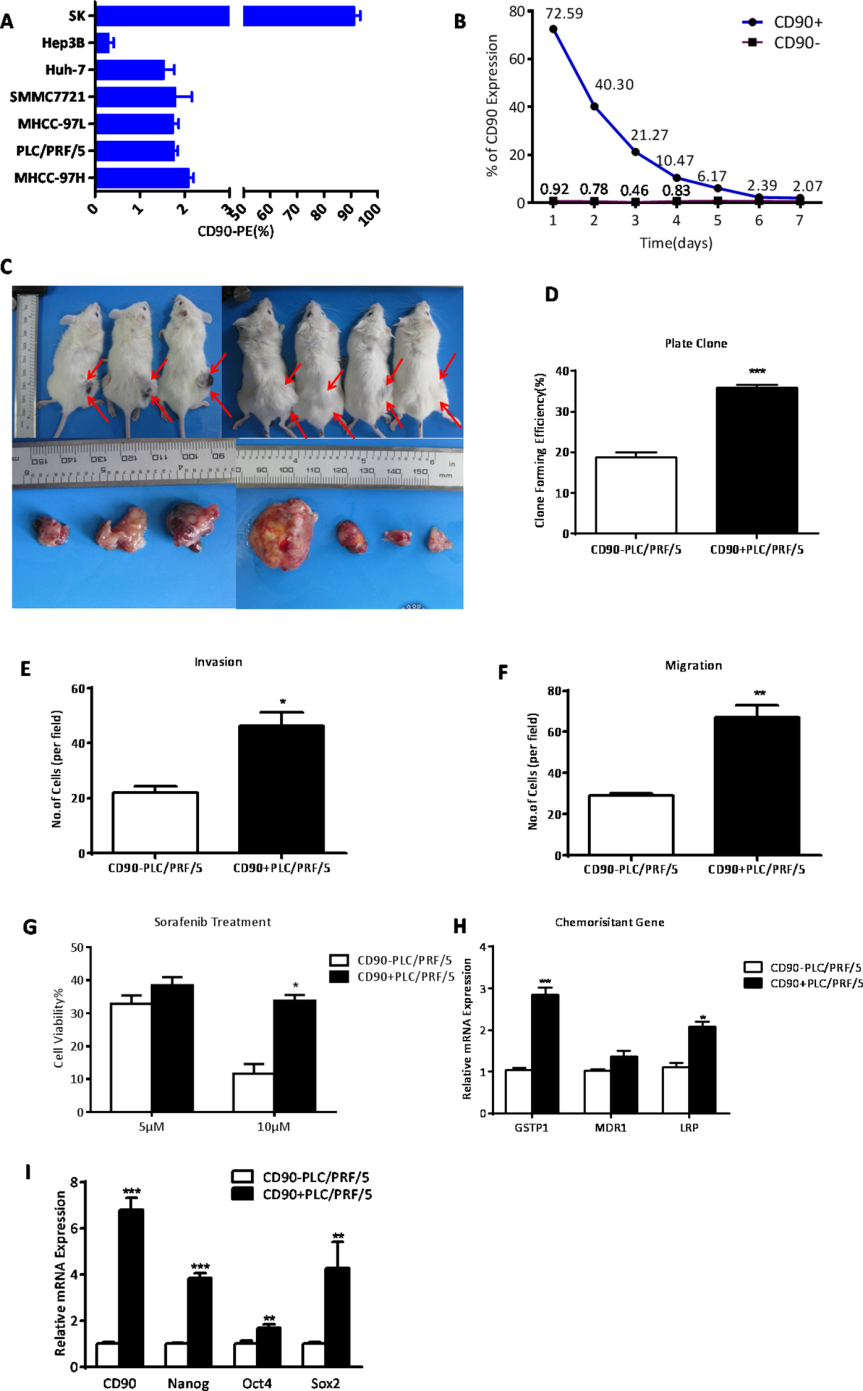
CD90^+^ HCC cells exhibited characteristics of cancer stem cells **A.** By FACS analysis, the HCC cell lines tested varied in CD90 expression levels. **B.** When CD90^+^ and CD90^−^ cells both isolated from PLC/PRF/5 cell line were separately cultivated in 10% serum-supplemented medium for one week, after which CD90 expression was analyzed in each population by FACS analysis. The proportion of CD90^+^ cells was dramatically declined and reverted almost to the presorting level after 6 days, whereas CD90^−^ cells retained low proportion even after 1 week. **C.** Efficiency of tumor formation of CD90^+^ cells isolated from PLC/PRF/5 cell line (7/9, as shown in Supplementary Table 1). Right flanks of mice were injected with CD90^+^ cells while left flanks were injected with CD90^−^ cells. Red arrows indicated the site of tumor formation. **D.** By colony formation assay on the sorted cells of PLC/PRF/5, it was demonstrated that CD90^+^ HCC cells had a significantly higher proliferation rate when compared with CD90^−^ cells (***P < 0.005, t test). **E, F.** Using trans-well migration and invasion assays, it was shown that the CD90^+^ cells sorted from PLC/PRF/5 cells had enhanced invasive (*P < 0.05, t test) and migratory (**P < 0.01, t test) properties in vitro. **G.** CD90^+^ and CD90^−^ cells derived from PLC/PRF/5 were treated with 5 or 10 μM sorafenib for 48 hours. Percentages of cell survival are shown (*P < 0.05, t test). **H.** Expression levels of drug-resistance relative genes were determined by qRT-PCR (*P < 0.05, **P < 0.01, t test). **I.** CD90^+^ cells from PLC/PRF/5 overexpressed several genes related to stemness when compared with CD90^−^ cells (**p < 0.01, ***P < 0.005, t test).

CD90 expression was examined by flow cytometry (FACS) analysis in a panel of HCC cell lines, including SK-hep1, Hep3B, Huh-7, SMMC7721, MHCC-97L, PLC/PRF/5, and MHCC-97H. Six of the cell lines expressed CD90 ranging from 0.3%±0.2% (Hep3B) to 2.08%±0.21% (MHCC-97H). SK-hep1 expressed CD90 at 91.1%±4.03% (Fig. 2A). Upon culture in serum-containing medium for 1 week, magnetic beads-sorted 72.59% CD90^+^ PLC/PRF/5 cells gradually decreased to the original proportion (about 1-2%). Whereas CD90^−^ cells failed to give rise to the high CD90 expression in the same culture condition, suggesting that CD90^+^ cells can differentiate into CD90^−^ cells in conventional culture conditions, but not vice versa (Fig. 2B). To investigate the tumor-initiating capacity of CD90^+^ cells in vivo, CD90^+^ and CD90^−^ cells were subcutaneously implanted in Severe Combined Immunodeficiency (SCID) mice (Fig. 2C, Supplementary Table 1). Our data showed that 2000 CD90^+^ cells isolated from both PLC/PRF/5 and Huh-7 could form tumors in 4 weeks, whereas 10,000 CD90^−^ cells could not form any tumor over the same period of time. Moreover, the efficiency of tumor formation by CD90^+^ PLC/PRF/5 and CD90^+^ Huh-7 were 7/9 (no. of mice with tumor formation/no. of mice injected) and 4/9, respectively, while CD90^−^ cells developed no tumors. Therefore, CD90^+^ cells showed significantly higher tumorigenic capacity than CD90^−^ cells. In addition, colony formation assay indicated that CD90^+^ cells were able to produce more and larger colonies than CD90^−^ cells (***P < 0.005, t test, Fig. 2D). Also, by transwell invasion and migration assay CD90^+^ cells displayed significantly higher invasive activity and migratory ability in vitro than CD90^−^ cells (*P < 0.05, **P < 0.01, t test, Fig. 2E, 2F).

Next, we tested whether CD90^+^ and CD90^−^ cells have different sensitivity to Sorafenib, which is a treatment for patients with advanced HCC. We found that CD90^+^ cells were more resistant to 10 μM Sorafenib than CD90^−^ cells by CCK8 toxicity assay (*P < 0.05, t test, Fig. 2G). In addition, multiple drug resistance genes, such as lung resistance protein (LRP), and glutathione S-transferases P1 (GSTP1) were found to be preferentially expressed in CD90^+^ cells by qRT-PCR, as compared with CD90^−^ cells (*P < 0.05, **P < 0.01, both t test, Fig. 2H). Using qRT-PCR analysis, we found that CD90^+^ cells purified from PLC/PRF/5 had a higher expression of stem-associated genes Nanog, Oct4 and Sox2 than that of CD90^−^ cells (*P < 0.05, **P < 0.01, ***P < 0.005, both t test, Fig. 2I). Together, these data demonstrated that CD90^+^ cells possessed a high capacity of tumorigenicity, invasion, metastasis, and resistance to chemotherapy, which was consistent with crucial hallmarks in the definition of CSCs.

### The activation of Notch signaling pathway enhanced the cancer stem cell features of CD90^−^ HCC cells

**Figure 3:**
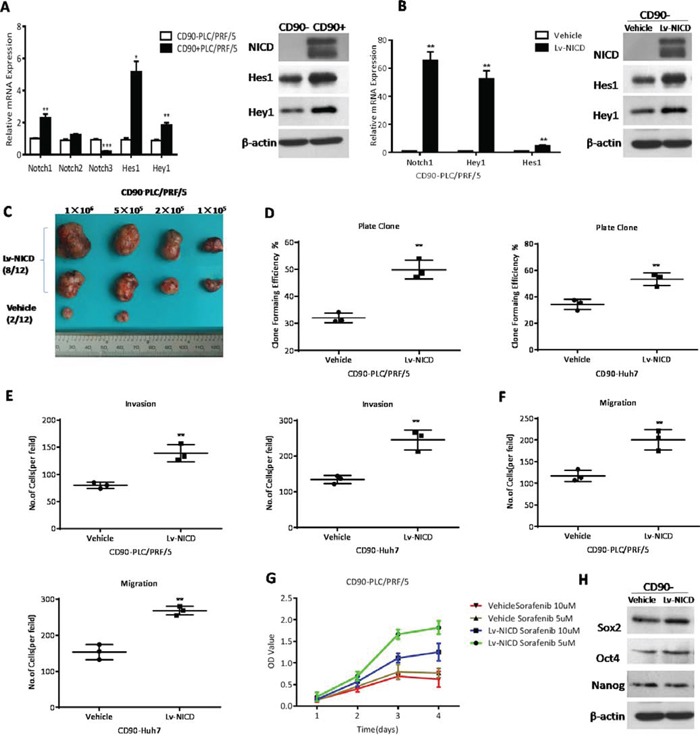
The activation of Notch signaling pathway enhanced the cancer stem cell features of CD90^−^ HCC cells **A.** CD90^+^ cells from PLC/PRF/5 overexpressed the core components of Notch pathway by qRT-PCR and WB when compared to CD90^−^ cells (*P < 0.05, **p < 0.01, ***P < 0.005, t test). **B.** Elevated expression of Notch (Notch1 and NICD), Hes1 and Hey1 by qRT-PCR and WB was observed after transinfection with Lv-NICD in CD90^−^ cells (**p < 0.01, t test). **C.** The efficiency of tumor formation of CD90^−^ PLC/PRF/5 cells after transinfection with Lv-NICD (8/12) or control lentivector (2/12, as shown in Supplementary Table 2). **D.** Colony formation efficiency of CD90^−^ cells from PLC/PRF/5 and Huh7 after overexpression of NICD (**p < 0.01, t test). **E, F.** Migratory and invasive activities of CD90^−^ cells from PLC/PRF/5 and Huh7 were determined by transwell assay (**p < 0.01, t test). **G.** CCK8 assays were used to measure the viability of CD90^−^ PLC/PRF/5 with empty vector or Lv-NICD after treatment of 5 and 10 μM sorafenib. **H.** CD90^−^ PLC/PRF/5 cells transinfected with Lv-NICD exhibited enhanced expression of Sox2 and Nanog by WB, vehicle as controls.

To examine whether Notch signaling was activated in CD90^+^ cells, we measured the expression levels of the core components by qRT-PCR and WB (Western blotting). We found elevated Notch1, NICD, and downstream target genes Hes1 and Hey1 in CD90^+^ cells, with CD90^−^ cells as controls (*P < 0.05, **P < 0.01, ***P < 0.005, both t test, Fig. 3A). In addition, Notch pathway was activated based on the high levels of Notch1 expression in HCC tissues (P = 0.0002, paired t test, Fig. 1B). Hence, we proposed Notch signaling was involved in the carcinogenesis of CD90^+^ cells and furthered our study to determine whether Notch signaling affect the tumorigenicity, invasion and metastatic abilities of CD90^+^ cells. CD90^−^ cells were transfected with Lv-NICD, and 96 hours later, expression levels of Notch1, NICD, Hes1, Hey1 were significantly increased as compared to the controls. These results suggested Notch signaling was activated by exogenous overexpression of NICD in CD90^−^ cells (Fig. 3B). To detect the ability of tumor formation, CD90^−^ cells with Lv-NICD were subcutaneously injected into SCID mice. After the activation of Notch pathway, 10^5^ CD90^−^ cells developed tumors, whereas tumor formation needed at least 5×10^5^ CD90^−^ cells transinfected with empty vector (Supplementary Table 2). The results indicated the tumor formation ability in vivo of CD90^−^ cells with NICD overexpression (8/12) was significantly enhanced, when compared with controls (2/12, Fig. 3C). Furthermore, elevated colony-forming capacity, invasive and migratory ability was confirmed in CD90^−^ cells isolated from PLC/PRF/5 and Huh-7 with NICD overexpression (**P < 0.01, both t test, Fig. 3D, 3E, 3F). At last, CD90^−^ cells transinfected with Lv-NICD were treated with 5μM or 10μM Sorafenib and exhibited increased chemoresistance. Higher expression level of stem-associated gene Sox2 was observed with activation of Notch pathway (Fig. 3G, 3H), and the expression levels of Nanog and Oct4 was invariant. Moreover, human recombinant protein Jagged1, as one of the ligands of Notch pathway, was always used to activate Notch signaling in vitro [19, 20]. The data showed activation of Notch pathway by Jagged1 could induce the cancer stem cell characteristics of CD90^−^ cells (Supplementary Fig. 1). Collectively, NICD overexpression in CD90^−^ cells could induce cancer stem cell features.

### The inhibition of Notch pathway attenuated the cancer stem cell properties of CD90^+^ cells

**Figure 4:**
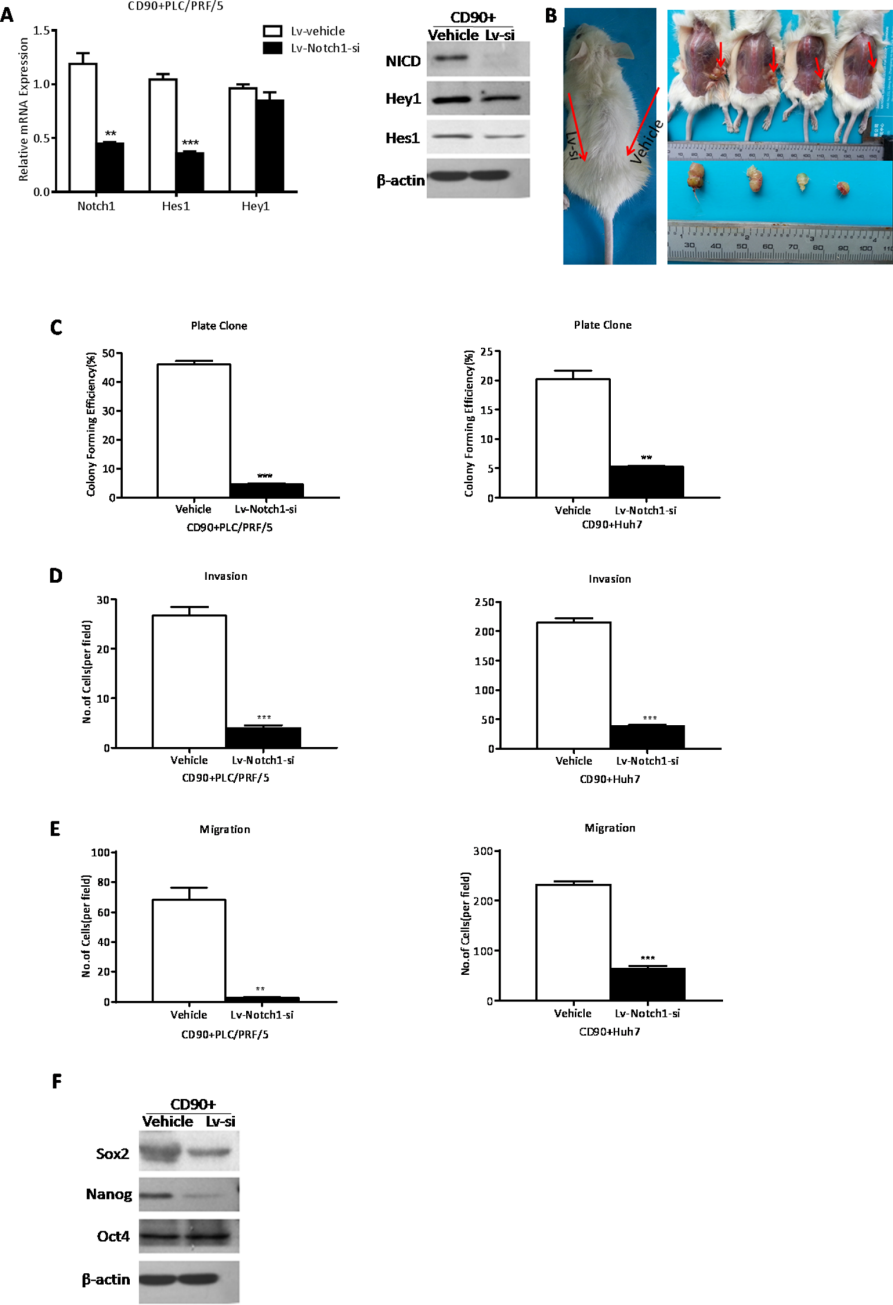
The inhibition of Notch pathway attenuated the cancer stem cell properties of CD90+ cells **A.** In CD90^+^ cells transinfected with Lv-Notch1-si the expression levels of NICD, Hes1 and Hey1 were decreased by qRT-PCR and WB (**p < 0.01, ***P < 0.005, t test). **B.** The efficiency of tumor formation of CD90^+^ PLC/PRF/5 cells after transinfection with Lv-Notch1-si (4/12) or control lentivector (0/12, as shown in Supplementary Table 2). Red arrows in the left graph indicated the sites of cells injection, while in the right graph indicated the site of tumor formation. **C.** By colony formation CD90^+^ cells transinfected with Lv-Notch1-si had a significantly lower proliferation rate when compared with CD90^−^ cells (**p < 0.01, ***P < 0.005, t test). **D, E.** The invasive (D) and migratory (E) activities of CD90^+^ cells from PLC/PRF/5 and Huh7 could be reduced after downregulation of NICD (**p < 0.01, ***P < 0.005, t test) **F.** Western blotting analysis of stem cell marker Sox2 and Nanog in CD90^+^ PLC/PRF/5 cells when attenuated the expression of NICD.

To further confirm whether NICD facilitates the stem cell behavior of CD90^+^ cells, Notch pathway in CD90^+^ cells was significantly inhibited through Lv-Notch1-si (**P < 0.01, ***P < 0.005, both t test, Fig. 4A) and showed decreasing tumorigenicity (Fig. 4B, Supplementary Table 2). In addition, CD90^+^ cells formed less colonies after Notch pathway was hindered, compared with controls (**P < 0.01, ***P < 0.005, both t test, Fig. 4C). Following the downregulation of NICD we found that CD90^+^ cells were significantly weakened the capacity of invasion (***P < 0.005, t test, Fig. 4D) and migration (**P < 0.01, ***P < 0.005, both t test, Fig. 4E), the expression level of stem cell-associated genes Sox2 and Nanog (Fig. 4F). Consistently, Notch pathway inhibited by RO4929097, the γ-secretase inhibitor, could also hinder the cancer stem cell features of CD90^+^ cells (Supplementary Fig. 2). In conclusion, the downregulation of Notch pathway diminished the cancer stem cell characteristics of CD90^+^ cells.

### The activation of Notch signaling promoted G1-S transition in the cell cycle phase of CD90^−^ HCC cells

**Figure 5:**
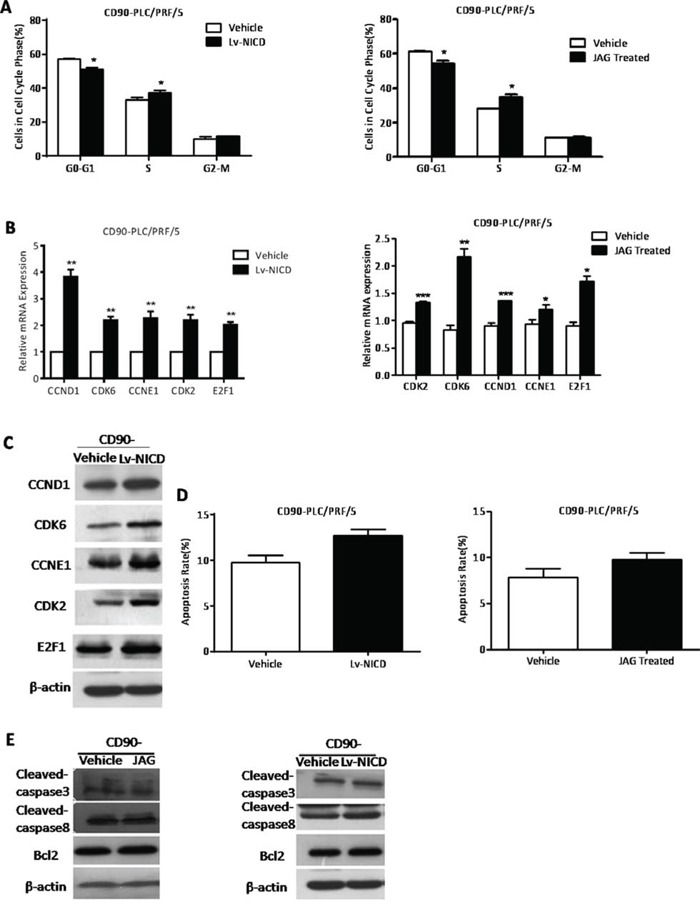
The activation of Notch signaling promoted G1-S transition in the cell cycle phase of CD90^−^ HCC cells **A.** By FACS analysis, CD90^−^ cells treated with Lv-NICD or Jagged1 exhibited a decreasing proportion of cells in G0-G1 phase and an increasing proportion of cells in S phase, compared with controls (*P < 0.05, t test). **B, C.** The detection of G1-S transition associated genes in CD90^−^ cells treated with Lv-NICD or Jagged1 by qRT-PCR and WB (*P < 0.05, **p < 0.01, ***P < 0.005, t test). **D, E.** By FACS analysis, the apoptosis rate of CD90^−^ cells after activation of Notch pathway was shown and the apoptosis-related genes were determined by WB.

After Notch signaling was activated in CD90^−^ cells treated with Lv-NICD or Jagged1, cells exhibited a decreasing proportion of cells in G0-G1 phase and an increasing proportion of cells in S phase (*P < 0.05, t test, Fig. 5A). No obvious differences were observed in G2-M phase. Through qRT-PCR and WB, we detected a series of cyclins and CDKs involved in G1-S transition that could be potential NICD targets. The data showed that NICD overexpression resulted in significant promotion in CylinD1 (CCND1), CyclinE1 (CCNE1), CDK2, CDK6 and E2F1 (*P < 0.05, **P < 0.01, ***P < 0.005, both t test, Fig. 5B, 5C). Apoptosis proteins (cleaved-caspase3 and cleaved-caspase8), and anti-apoptosis gene (Bcl2), are used to evaluate the degree of apoptosis. The analysis of FACS and WB showed us the apoptosis rate of CD90^−^ cells had not been changed with the activation of Notch signaling (Fig. 5D, 5E). Therefore, the activation of Notch signaling induced G1-S transition in the cell cycle phase of CD90^−^ cells.

### The inhibition of Notch signaling suppressed G1-S transition in the cell cycle phase and promoted apoptosis of CD90^+^ HCC cells

**Figure 6:**
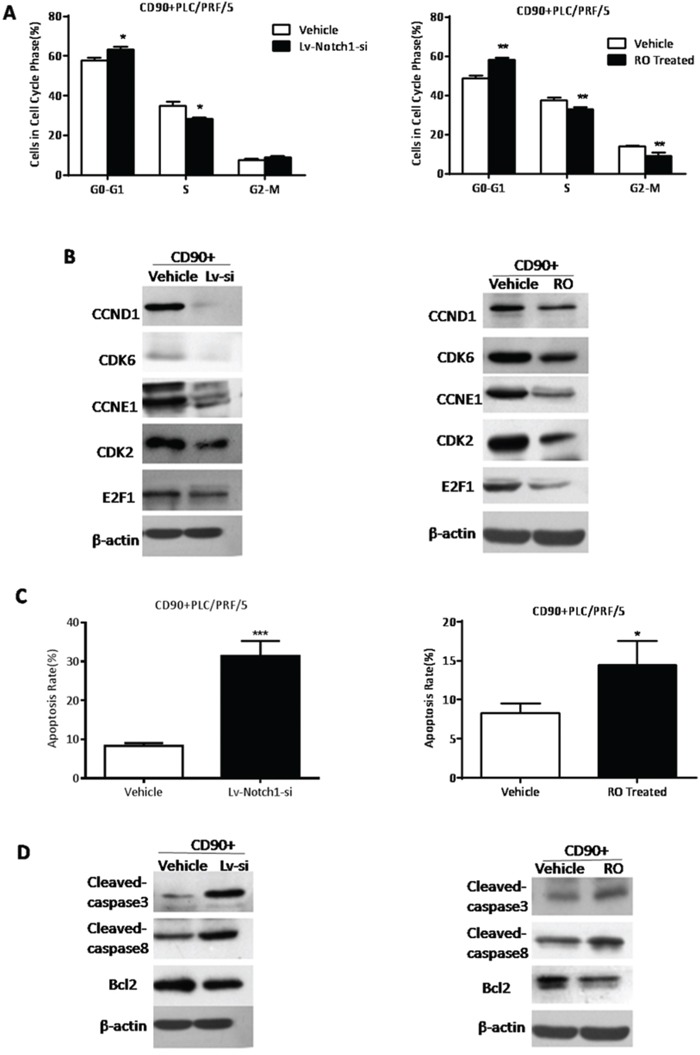
The inhibition of Notch signaling suppressed d G1-S transition in the cell cycle phase and increased apoptosis of CD90^+^ HCC cells **A.** CD90^+^ cells treated with RO4929097 or Lv-Notch1-si exhibited an increasing proportion of cells in G0-G1 phase and decreasing proportion of cells in S phase, compared with both controls(*P < 0.05, **p < 0.01, t test). **B.** By WB the detection of G1-S transition associated genes in CD90^+^ cells while knockdown of Notch expression. **C.** The apoptosis rate of CD90^+^ cells after the inhibition of Notch pathway was detected by FACS analysis (*P < 0.05, ***P < 0.005, t test). **D.** The apoptosis protein cleaved-caspase3 and cleaved-capase8 was elevated, whereas anti-apoptosis gene Bcl2 was reduced in CD90^+^ cells treated with RO4929097 or Lv-Notch1-si.

CD90^+^ cells exhibited ascending proportion of cells in G0-G1 phase and an decreasing proportion of cells after Notch signaling was inhibited by RO or Lv-Notch1-si (*P < 0.05, **P < 0.01, both t test, Fig. 6A). Western Blotting also demonstrated that a panel of genes involved in G1-S transition decreased at protein levels (Fig. 6B). In CD90^+^ cells with knockdown of NICD the apoptosis rate by FACS analysis was increased (*P < 0.05, ***P < 0.005, both t test, Fig. 6C). Apoptosis associated proteins cleaved-caspase3, and cleaved-caspase8 presented a rising trend, and anti-apoptotic gene Bcl-2 decreased on the contrary (Fig. 6D). Hence, inhibition of Notch signaling suppressed G1-S transition in the cell cycle phase of CD90^+^ cells and promoted apoptosis.

## DISCUSSION

Consistent with Sukowati et al.'s study [21], we also observed that only a minority of cells in HCC tissues demonstrateded CD90 expression, whereas CD90 expression was hardly observed in the non-tumor liver tissues. Of note, strong positive overexpression of CD90 was significantly correlated with venous infiltration in HCC patients. Those HCC patients could be more likely to develop HCC metastasis and poor prognosis. But it has not ever been proposed that the underlying biological mechanism about CD90 in HCC [4, 22]. Our vitro experiments showed that CD90^+^ cells derived from HCC cell lines possessed a high capacity of tumorigenicity, invasion, metastasis, and resistance to chemotherapy. Therefore, our study demonstrated that CD90^+^ HCC cells displayed the capacity to initiate and sustain tumor growth, leading eventually to cancer metastasis.

Recent studies indicated that the Notch pathway promoted the tumorigenesis and radioresistance in glioma stem cells [23, 24]. And the Notch pathway was important in maintaining the cancer stem cell population in pancreatic cancer [25]. However, until now there are still no reports about the involvement in the Notch pathway and CD90^+^ HCC CSCs. Our study showed Notch1 in HCC tissues expressed much higher than non-HCC tissues. And the Notch1 pathway was activated in CD90^+^ CSCs indicating Notch pathway may contribute to the carcinogenesis of HCC. Moreover, the activation of Notch signaling pathway enhanced the cancer stem cell features of CD90^−^ HCC cells, and the inhibition of Notch pathway attenuated the cancer stem cell properties of CD90^+^ cells. Taken together, the Notch pathway could promote the cancer stem cell characteristics of CD90^+^ CSCs and may be an attractive therapeutic target for HCC patients.

Maintenance of pluripotency in embryonic stem cells is governed by the expression of the core transcription factors Nanog, Oct4, and Sox2, as well as a variety of other factors [26]. Sox2 and Nanog are also involved in the maintain CSCs of solid tumors [27, 28]. In our research, we found that the expression levels of Sox2 and Nanog in HCC tissues were much higher than non-tumors and positively related with NICD expression in CD90^+^ CSCs. This result suggested that Sox2 and Nanog probably contributed to the cancer stem cell characteristics of CD90^+^ CSCs through Notch pathway. According to Xu et al.'s study, Sox2 could at least partially control Notch pathway through binding to Notch1 and Notch2 regulatory regions in lung adeno carcinoma [29]. Nanog, Oct4, and Sox2 have been shown to repress the expression of developmental genes while modulating their own expression levels by binding to each other's promoter regions [30, 31]. However, the role which Sox2 and Nanog played in Notch signaling in CD90^+^ CSCs, have not been reported until now and still needed our further investigation.

In our previous study, Notch signaling could induce apoptosis and block G1-S transition to interrupt the occurrence and development of HCC [32, 33]. This study demonstrated that CD90^+^ cells was inhibited in the G1-S transition and induced apoptosis while Notch pathway was disrupted. While CD90^−^ cells were accelerated in G1-S transition after activation of Notch signaling. But the apoptosis rate of CD90^−^ cells had not been increased with the activation of Notch signaling. Probably because the PLC/PRF/5 cell line was in very good condition and the apoptosis rate could be quite low.

Taken together, we may conclude that CD90 is a prognostic marker for HCC and characterize CD90^+^ cells as hepatic CSCs. Furthermore, our data established that Notch pathway stimulated the cancer stem cell characteristics of CD90^+^ cells through cell cycle and apoptosis.

## MATERIALS AND METHODS

### Clinical specimens

HCC and non-HCC samples were obtained with informed consent from patients who had undergone radical resection at the Hepatic Surgery in Tongji Hospital (Wuhan, China). The tissue acquisition procedures were approved by the ethics committee of Tongji Hospital. A total of 31 pairs of formalin-fixed and paraffin-embedded human HCC and corresponding non-HCC tissues, for immunhistochemistry were obtained from 2013 to 2014. The staining intensity was scored on a scale of 0 to 3 as negative (0), weak (1), medium (2) or strong (3). The extent of the staining, defined as the percentage of positive staining areas of tumor cells in relation to the whole tumor area, was scored on a scale of 0 to 4: 0(0%), 1 (1–25%), 2 (26–50%), 3 (51–75%) and 4 (76–100%). An overall protein expression score (overall score range, 0–12) was calculated by multiplying the intensity and positivity scores. For statistical analysis, a final staining score greater than zero was considered to be positive sample. IHC staining score ≥10 was defined as high expression.

### Cell culture

Human HCC cell lines (SK-hep1, Hep3B, Huh-7, SMMC7721, MHCC-97L, PLC/PRF/5 and MHCC-97H) were purchased from the Shanghai Cell Collection (Shanghai, China). All cell lines were cultured in Dulbecco's modified Eagle's medium (DMEM, GIBCO) supplemented with 10% fetal bovine serum (FBS, GIBCO) at 37°C, 5% CO2 condition.

### Flow cytometry analysis

Adherent and suspended cells were dissociated into single cells and labeled with PE-conjugated CD90 human antibody (clone 5E10, BD PharMingen) at 4°C for 15 minutes. Concentrations of antibodies were used according to the manufacturers' recommendations. The stained cells were analyzed with the FACS Calibur machine and CellQuest software (BD Biosciences).

### Isolation of CD90^+^ and CD90^−^ populations by magnetic bead cell sorting

For magnetic cell sorting, cells were labeled with CD90 microbeads human antibody (Miltenyi Biotec, Germany). Sorting was carried out with the Miltenyi Biotec MidiMACS Starting Kit according to the manufacturer's instructions. Magnetic separation was performed up to three times to obtain a CD90^+^ population more than 70% pure. Aliquots of CD90^+^ and CD90^−^ sorted cells were evaluated for purity with a FACS Calibur machine and CellQuest software (BD Biosciences).

### Tumor formation assay

NOD/SCID mice at age of 3-5 weeks, male, were maintained in pathogen-free conditions at animal facility of Tongji Medical College and received humane care according to the criteria outlined in the “Guide for the Care and Use of Laboratory Animals” prepared by the National Academy of Sciences. The different numbers of CD90^+^ and CD90^−^ cells were resuspended in serum-free medium and were mixed with Matrigel at the ratio of 1:1. The cells were subcutaneously injected into SCID mice. Tumor formation was evaluated regularly after injection by palpation of injection sites.

### Lentiviral-based transfection into HCC cells

For suppression or activation of the Notch pathway in sorted HCC cells, lentiviral particles (Genechem, Shanghai) expressing Notch1-siRNA or NICD were used in our study. The siRNA sequence targeted Notch1 was listed as follows: 5′-GGAGCATGTGTAACATCAA-3′. For optimization of transfection conditions with lentiviral vectors, HCC cells were infected with Lv-NICD or Lv-Notch1-si at different of multiplicity of infection (MOI) for 12 hours in the presence of 5μg/ml of polybrene. Two days after infection, expression of green florescence protein was measured by FACS analysis. Our data showed that infection of sorted cells at MOI of 10 resulted in more than 90% of efficiency of infection without damaging cells.

### Colony formation assay

Cells were seeded at a density of 1,000 cells per well in six well plates and allowed to grow for six days. Clones were fixed by 4% methanol and dye with Giemsa (Sigma Aldrich) and clone (>50 cells) numbers were counted microscopically.

### Migration and invasion assays

The migration assay was performed as described [11]. The invasion assay was performed as described [10]. Photographs of three randomly selected fields of the fixed cells were captured and cells were counted. The experiments were repeated independently three times.

### CCK8 toxic assays

The sensitivity of CD90^+^ and CD90^−^ cells to chemotherapeutic drugs sorafenib was measured by CCK8 assay. Briefly, cells were seeded in 96-well plates and then were treated with various concentrations (5 μM or 10 μM) of sorafenib (Sigma-Aldrich) at second day, co-incubated for 24h, or 36h or 48h. After changing to fresh medium without sorafenib, cells were then added CCK8 reagent (Promoter, Wuhan) to each well according to the manufacturer's instructions. Absorbance was measured at 490 nm.

### Western blot

For immunoblotting, equal amounts of proteins (30∼80μg) were separated on 10%–12% SDS-PAGE and were electrophoretically transferred onto PVDF membranes (Millipore), which were blocked in TBST containing 5% defatted milk for 1 hour at RT and then blotted with antibody overnight at 4°C: anti-Nanog (1:1000, Cell signaling), anti-Oct4 (1:1000, Cell signaling), anti-Sox2 (1:1000, Cell signaling), anti-CyclinD1 (1:1000, Cell signaling), anti-CyclinE1 (1:1000, Cell signaling), anti-E2F1 (1:1000, Cell signaling), anti-CDK2 (1:1000, Cell signaling), anti-CDK6 (1:1000, Cell signaling) and anti-β-actin (1:1000, Cell signaling). After being washed with TBST and incubation with either anti-rabbit or anti-mouse horseradish peroxidase-conjugated secondary antibody (1:5000, Promoter Wuhan) in TBST containing 5% nonfat milk, immune complexes were visualized using the Beyo ECL Plus.

### Immunohistochemistry

Immunohistochemical staining was performed on formalin fixed, paraffin-embedded tissue sections using the labeled streptavidin biotinperoxidase complex method. The procedure for IHC Antibody used for detection of Nanog, Sox2, Oct4 and Notch1 was from Cell Signaling. The staining intensity and the extent of staining were scored as described [11]. For statistical analysis, a final staining score greater than zero was considered to be positive sample.

### Quantitative RT–PCR

Total RNA was extracted from cancer cells using RNAiso Plus (Takara, Japan). The cDNA synthesis was performed according to the manufacturer's instructions (Takara, PrimeScript RT Master Mix). Quantitative PCR was performed by SYBR Premix Ex TaqTM (Takara, DRR081A) using a Stepone Real-Time System (Bio-rad). PCR reaction conditions for all assays were 94°C for 30 seconds, followed by 40 cycles of amplification (94°C for 5 seconds, 60°C for 30 seconds and 72°C for 30 seconds). β-actin mRNA was used to normalize RNA inputs. RT–PCR primers were as described in Supplementary Table 3.

### Statistical analysis

All data are presented as mean±standard deviation. Student's t-test was used for two group's comparison. Analysis of variance was used for clinical statistical analyses. In all cases, P < 0.05 was considered with statistical significant.

## SUPPLEMENTARY FIGURES AND TABLES


