# Supplementary material for: The Notch pathway promotes the cancer stem cell characteristics of CD90+ cells in hepatocellular carcinoma
Source: Oncotarget. 2015 Dec 19;7(8):9525–37. doi: 10.18632/oncotarget.6672 (PMC4891057; doi:10.18632/oncotarget.6672)
Supplement: Supplementary file 1 [file oncotarget-07-9525-s001.pdf]

SUPPLEMENTARY FIGURES AND TABLES

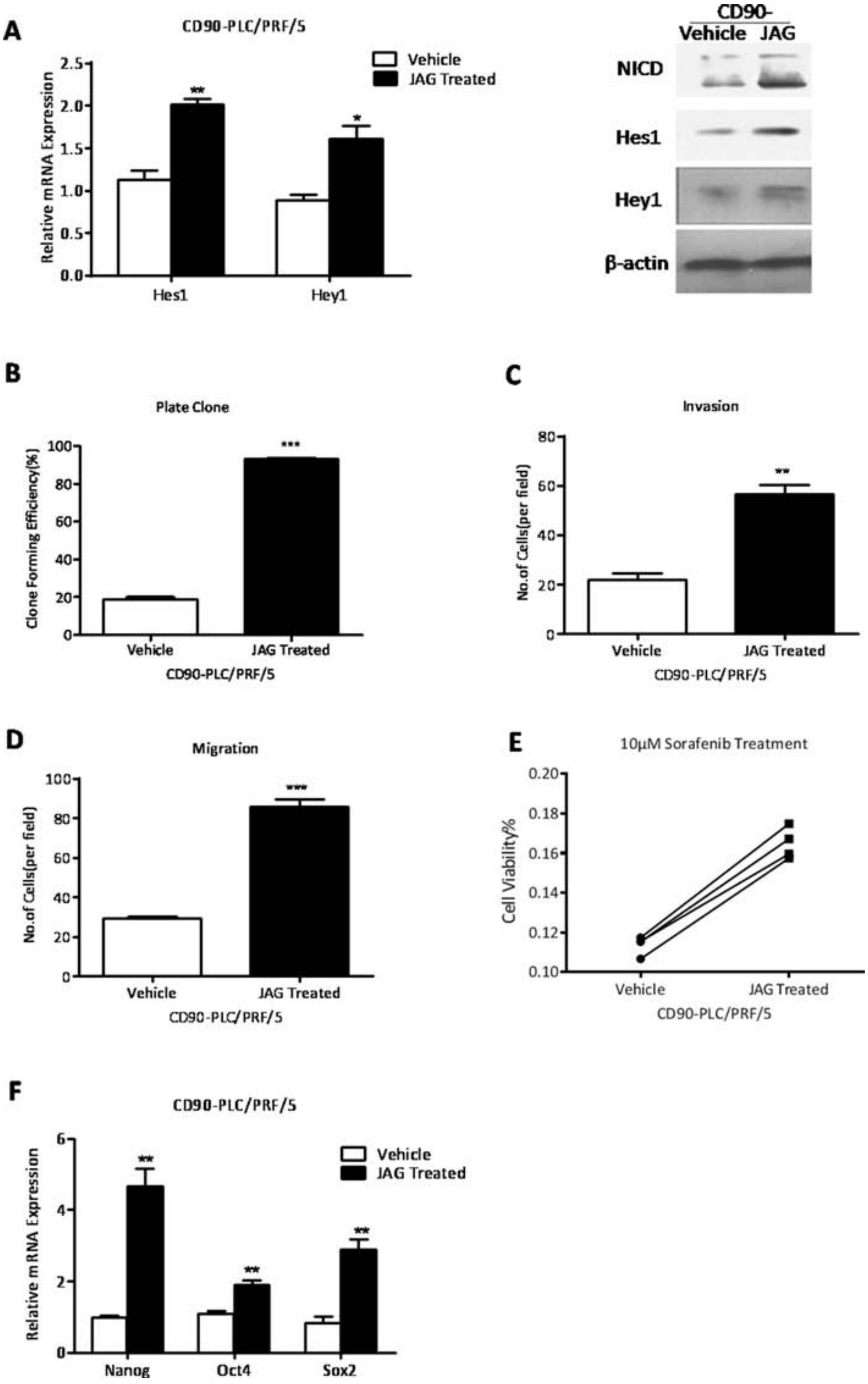

Supplementary Figure S1: CD90- cells possessed characteristics of cancer stem cells after Notch signaling activated by Jagged1. The core components of Notch signaling in CD90- cells could be increased by human recombinant protein Jagged1 (Supplementary Fig. 1A). With the overexpression of NICD, we found the capacity of colony formation, invasion and migration in CD90- cells was elevated following activation of Notch pathway (Supplementary Fig. 1B, 1C, 1D). Treated with 10 μM Sorafenib CD90- cells displayed promoted viability following NICD overexpression (Supplementary Fig. 1E). In addition, Nanog, Sox2 and Oct4 were also enhanced in expression levels detected by qRT-PCR (Supplementary Fig. 1F).

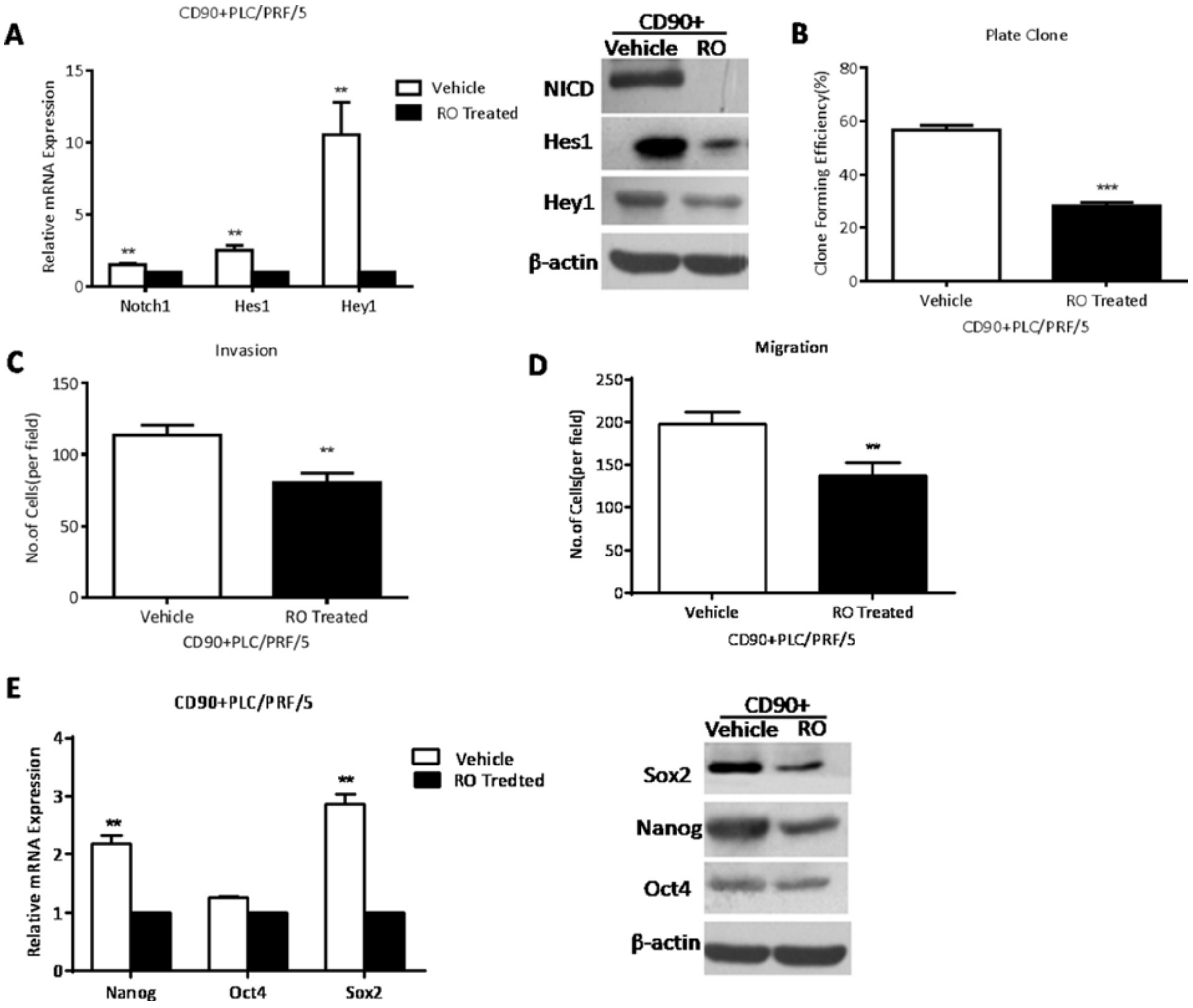

Supplementary Figure S2: The inhibition of Notch pathway by RO4929097 attenuated cancer stem cell characteristics of CD90$^+$ cells. RO4929097, as the γ-secretase inhibitor, could downregulate the Notch pathway in CD90$^+$ cells as shown in Supplementary Fig. 2A. Accompanied with decreasing NICD, CD90$^+$ cells showed presented the declining capacity of colony formation, migration and invasion (Supplementary Fig. 2B, 2C, 2D), and embryonic stem cell-associated markers (Nanog, Sox2) in CD90$^+$ cells was also reduced after treated with RO4929097 (Supplementary Fig. 2E). However, there was no change in expression of Oct4.

Supplementary Table S1: Tumorigenic capacity of $CD90^+$ cells from HCC cell lines

Cell Lines	Phenotypes	No. of Injected Cells	Tumor Incidence*	Latency(days)**
PLC/PRF/5	$CD90^+$	2×10^3	2/3	20
		1×10^4	2/3	15
		5×10^4	3/3	7
	$CD90^-$	2×10^3	0/3	-
		1×10^4	0/3	-
		5×10^4	0/3	-
Huh-7	$CD90^+$	1×10^3	0/3	-
		2×10^3	1/3	10
		1×10^4	3/3	8
	$CD90^-$	1×10^3	0/3	-
		2×10^3	0/3	-
		1×10^4	0/3	-

*No. of mice with tumor formation/no. of mice injected.
**Approximate no. of days from cells injection to appearance of the tumor.

Supplementary Table S2: Tumorigenic capacity of $CD90^+/CD90^-$ cells from PLC/PRF/5 after the inhibition or activation of Notch pathway

Phenotypes	No. of Injected Cells	Tumor Incidence*	Latency(days)**
$CD90^+$+Vehicle	2×10^3	0/3	-
	1×10^4	0/3	-
	5×10^4	1/3	36
	1×10^5	3/3	28
$CD90^+$+Lv-Notch1-si	2×10^3	0/3	-
	1×10^4	0/3	-
	5×10^4	0/3	-
	1×10^5	0/3	-
$CD90^-$+Vehicle	1×10^5	0/3	-
	2×10^5	0/3	-
	5×10^5	1/3	70
	1×10^6	1/3	63
$CD90^-$+Lv-NICD	1×10^5	2/3	67
	2×10^5	2/3	56
	5×10^5	2/3	44
	1×10^6	2/3	37

*No. of mice with tumor formation/no. of mice injected.
**Approximate no. of days from cells injection to appearance of the tumor.

Supplementary Table S3: Primer sequences for quantitative RT-PCR analysis

Gene	GENE ID.	Primer sequences (5'-3')	Product length
Sox2	6657	F:GCCCTGCAGTACAACTCCAT<br>R:GACTTGACCACCGAACCCAT	110
Oct4	5460	F:CTTGAATCCCGAATGGAAAGGG<br>R:GACTTGACCACCGAACCCAT	111
Nanog	79923	F:GTCCCAAAGGCAAACAACCC<br>R:GCTGGGTGGAAGAGAACACA	108
CD90	7070	F:ACTGCCGCCATGAGAATACC<br>R:CTGGTGAAGTTGGTTCGGGA	136
MDR1	5243	F:CCCATCATTGCAATAGCAGG<br>R:GTTCAAACTTCTGCTCCTGA	157
GSTP1	2950	F:TGGTGGACATGGTGAATGAC<br>R:ATCTGGTCTCCCACAATGAAG	181
LRP	9961	F:GGATGTCAAGACCGGAAAGGT<br>R:TCTTTCTCCCACGGACTTCGT	80
Hes1	3280	F:GCTAAGGTGTTTGGAGGCT<br>R:CCGCTGTTGCTGGTGTA	122
Notch1	4851	F:CCGCAGTTGTGCTCCTGAA<br>R:ACCTTGGCGGTCTCGTAGCT	109
Hey1	23462	F:GTTCGGCTCTAGGTTCCATGT<br>CGTCGGCGCTTCTCAATTATTC	88
β-actin	60	F:GTTGCGTTACACCCTTTCTTG<br>R:GACTGCTGTCACCTTCACCGT	157
CyclinD1	595	F: GCTGCGAAGTGGAAACCATC<br>R: CCTCCTTCTGCACACATTTGAA	135
CyclinE1	898	F:GCCAGCCTTGGGACAATAATG<br>R:CTTGCACGTTGAGTTTGGGT	104
CDK2	1017	F:GTACCTCCCCTGGATGAAGAT<br>R:CGAAATCCGCTTGTTAGGGTC	75
CDK6	1021	F:CCAGATGGCTCTAACCTCAGT<br>R:AACTTCCACGAAAAAGAGGCTT	152
E2F1	1869	F:ACGTGACGTGTCAGGACCT<br>R:GATCGGGCCTTGTTTGCTCTT	146

F: Forward; R: Reverse.
